# Comparison of proximal gastrectomy with double-flap technique and double-tract reconstruction for proximal early gastric cancer: a meta-analysis

**DOI:** 10.1007/s13304-023-01638-w

**Published:** 2023-09-20

**Authors:** Qiao-zhen Huang, Peng-cheng Wang, Yan-xin Chen, Shu Lin, Kai Ye

**Affiliations:** 1 Department of Surgery, The Second Affiliated Hospital, Fujian Medical University, No.34 North Zhongshan Road, Quanzhou, 362000 Fujian China; 2 Centre of Neurological and Metabolic Research, The Second Affiliated Hospital of Fujian Medical University, No.34 North Zhongshan Road, Quanzhou, 362000 Fujian China; 3 Group of Neuroendocrinology, Garvan Institute of Medical Research, 384 Victoria St, Sydney, Australia

**Keywords:** Proximal gastric cancer, Proximal gastrectomy, Double-tract reconstruction, Double-flap technique

## Abstract

Surgical resection is the main treatment for proximal gastric cancer, but there is no consensus on its reconstruction. We carried out a meta-analysis to evaluate the effects of double-tract reconstruction (DTR) and double-flap technique (DFT) on postoperative quality of life in patients with proximal gastric cancer. Systematic searches of PubMed, Web of Science, EBSCO, and the Cochrane Library were performed. Literature for the last 5 years was searched without language restrictions. The cutoff date for the search was 12 April 2023. Literature and research searches were conducted independently by two researchers and data were extracted. Statistical analyses were performed using Review Manager (Revman) 5.4 software. Fixed models were used when heterogeneity was small and random-effects models were used for meta-analysis when heterogeneity was large. The study was registered with PROSPERO, CRD 42023418520. Surgical time was significantly shorter in the DTR group than in the DFT group (*P* = 0.03). There were no significant differences between DFT and DTR in terms of age, gender, pathological stage, preoperative body mass index, surgical bleeding, and perioperative complications. There was no statistically significant difference between the two groups in terms of reflux esophagitis and PPI intake, but DFT was superior to DTR in weight improvement at 1 year after surgery (*P* < 0.0001). Compared with DTR, DFT reconstruction is more demanding and time-consuming, but its postoperative nutritional status is better, so it should be the first choice for GI reconstruction in most patients with early proximal gastric cancer. However, DTR should be the best choice for patients who have difficulty operating.

**Supplementary Information:**

The online version contains supplementary material available at 10.1007/s13304-023-01638-w.

## Introduction

Gastric cancer is one of the top five cancers in the world. Although the overall incidence of gastric cancer has been decreasing in recent decades, the incidence of proximal gastric cancer is on the rise, especially in Asian countries [1]. Surgery is the main treatment for early proximal gastric cancer [2]. Total gastrectomy (TG) was once considered the standard surgical procedure for the treatment of proximal gastric cancer, but it may lead to poorer long-term nutritional problems [3]. As the survival rate of gastric cancer gradually improves, more and more surgeons are focusing on the quality of life of postoperative patients. Proximal gastrectomy (PG) preserves the physiological and storage functions of the stomach and has advantages in maintaining postoperative nutritional status and body weight [4, 5]. Currently, the main types of digestive tract reconstruction are double-tract reconstruction (DTR), jejunal implantation, jejunal pouch implantation, double-flap anastomosis (DFT), and fundoplication. Each of these reconstruction methods has advantages and disadvantages. Due to the lack of multi-center, large-sample studies, there is no evidence as to which reconstruction method is the best reconstruction strategy [6]. Reflux esophagitis and anastomotic stenosis after PG are important factors affecting patients’ postoperative quality of life. How to minimize these two complications is also a future endeavor for surgeons [7].

DFT is an anastomosis method with anti-reflux mechanism, but anastomotic stenosis is one of its main complications [8]. DTR is effective for reconstruction after proximal gastrectomy and has a low incidence of reflux esophagitis [3]. DTR and DFT can reduce the incidence of postoperative complications to some extent [9, 10]. The aim of this study was to compare the clinical efficacy and postoperative quality of life of DTR and DFT, and to provide guidance for the choice of digestive tract reconstruction after PG.

## Materials and methods

**Fig. 1:**
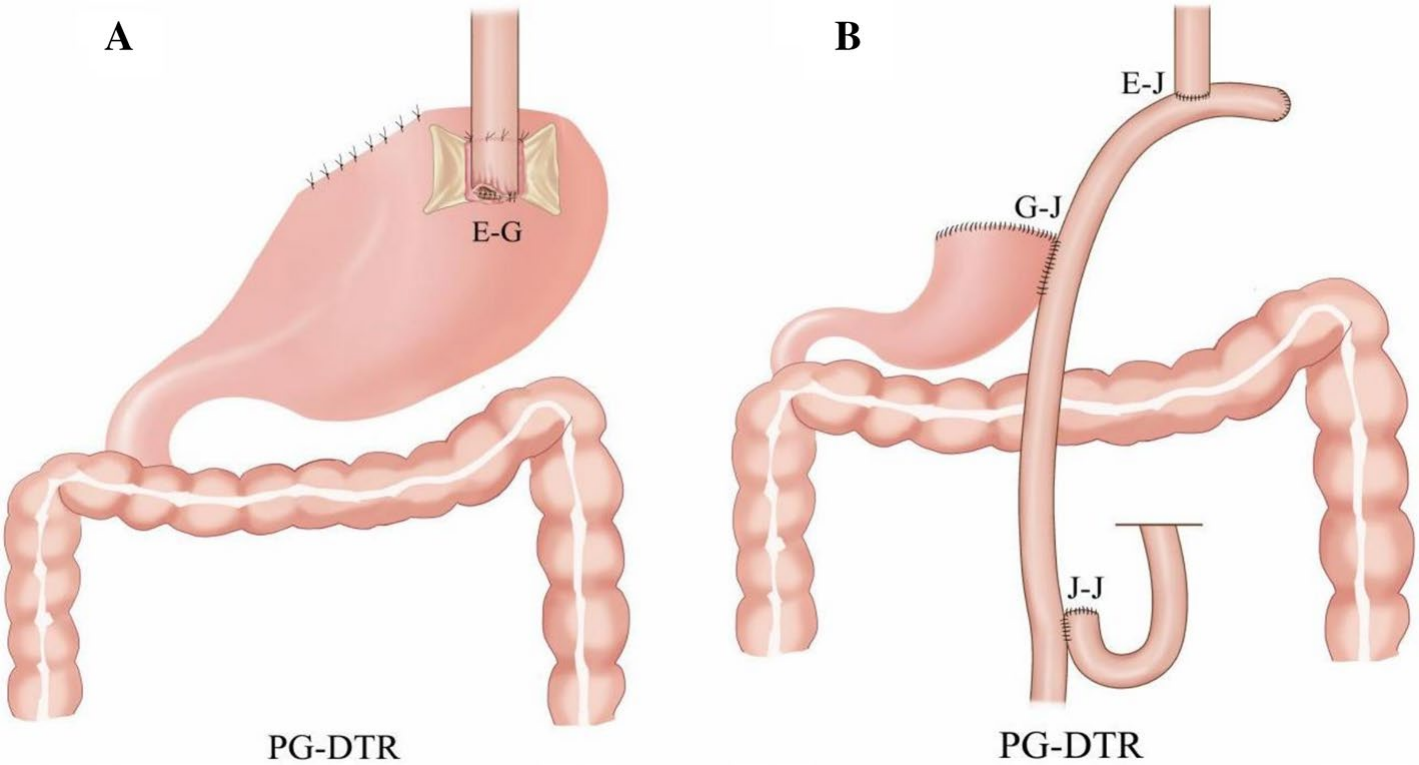
Digestive tract reconstruction after PG and image. **A** Proximal gastrectomy with double-flap technique; **B** proximal gastrectomy with double-tract reconstruction

The diagram of digestive tract reconstruction after PG is shown (Fig. 1).

### Search strategy

This study was based on the PRISMA 2020 Statement: Guidelines for Updating Systematic Review Reports [11]. A systematic search of PubMed, Web of science, EBSCO, and the Cochrane Library was conducted on April 12, 2023. Literature from the last 5 years was searched without language restrictions. Terms in the title and abstract were searched (‘proximal gastrectomy’ OR ‘partial gastrectomy’ OR ‘PG’ OR ‘cancer of the cardia’ OR ‘adenocarcinoma of esophagogastric junction’ OR ‘AEG’) AND (‘double flap technique’ OR ‘Kamikawa’ OR ‘Double-tract reconstruction’). We screened the titles and abstracts of all articles to select articles describing PG reconstruction methods for proximal gastric cancer and adenocarcinoma of the esophagogastric junction (AEG). To avoid missing any potential studies, we manually reviewed the references included in the literature. Full-text articles initially included in the study were screened. Literature retrieval and research selected two authors to independently review and extract data from each study, compare the search results, and resolve any differences through further discussion.

### Inclusion and exclusion criteria

Inclusion criteria: (1) clinically and pathologically confirmed as upper gastric cancer or Siwet II/III AEG; (2) cTNM stage I or II; (3) comparative studies of PG-DTR and PG-DFT; (4) the outcomes including basic information, perioperative status, and 1-year follow-up.

Exclusion criteria: (1) PG-DFT and PG-DTR reconstruction methods were not included in this study; (2) conference abstracts, case reports, reviews, commentaries, letters, meta-analyses, or animal studies; (3) patients with serious underlying medical conditions that may affect treatment selection and outcomes.

### Study selection

A total of 1646 articles were retrieved from PubMed, Embase, and Cochrane Library. Five studies that met the inclusion criteria were finally included in this study. The flow screening process is shown in Fig. 1. The characteristics of the included studies are shown in Table 1. A total of 409 patients were included in our analysis: 196 patients were treated with PG-DFT; 213 patients were treated with PG-DTR. Data extraction was performed independently by two authors (WPC and HQZ). When there was a difference of opinion, the problem was solved by a third researcher (CYX).

**Table 1:** The characteristics and NOS scored of the included five studies

References	Country	Total cases (DFT/DTR)	Tumor location	Stage	Operative procedures	Gender (M/F)	NOS scores
Yu et al. [17]	Korea	18/51	Upper-third stomach	I	L and O	50/19	8
Nishimura et al. [13]	Japan	35/8	Upper third of the stomach or gastroesophageal cancer located within 2 cm above the esophagogastric junction	I	L	39/4	9
Wei et al. [15]	China	35/33	Upper third of the stomach or Siewert II/III AEG	I, II	L	57/11	8
Li et al. [16]	China	72/107	Siewert II/III AEG	I, II	L and O	138/41	8
Saze et al. [14]	Japan	36/14	Upper third of the stomach	I, II	L and O	37/13	9

*L* laparoscope, *NOS* Newcastle–Ottawa Scale, *O* open, *DTR* double-tract reconstruction, *DFT* double-flap technique

### Data extraction

Data refinement included: (1) research background (first author’s surname, publication year, research design, nationality and number of patients in each group); (2) cohort characteristics (reconstruction method, average age of subjects, sex ratio); (3) perioperative outcomes (operation time, surgical bleeding); (4) postoperative complications (reflux esophagitis, PPI intake); (5) nutritional status 1 year after operation (BMI, hemoglobin (Hb) level).

The primary outcomes of this study were postoperative nutritional status, defined primarily as BMI or Hb level at 12 months postoperatively and reflux esophagitis at 12 months postoperatively. Secondary outcomes were surgical outcome, including operation time, surgical bleeding, and total perioperative complications.

### Study quality assessment

The assessment for inclusion in this study was done independently by two researchers. In case of disagreement, the decision will be made after discussion. The Newcastle–Ottawa Scale (NOS) was used to evaluate the quality of the controlled clinical trial (CCT). The scale consisted of study population selection (4 items), comparability between groups (2 items), and outcome indicators (3 items). Each score is 1 point, more than 6 points for a high-quality CCT, and up to 9 points [12].

### Assessment of publication bias across the included studies

The funnel plot of the standard error of the total complication rate was used to evaluate the publication bias of this map. In the absence of publication bias, it is assumed that high-precision studies will be drawn near the average (vertical line), and low-precision studies will be evenly distributed on both sides of the vertical line to form a funnel-shaped distribution.

### Statistical analysis

Statistical analyses were performed using Review Manager 5.4 software. Continuous variables were expressed as mean ± standard deviation (SD), or median and normal range. Categorical variables were expressed as number and percentage. A *p* value less than 0.05 was considered significant. Clinical outcomes and complications were analyzed using mean weight difference (WMDs), odds ratio (ORs), and 95% confidence interval (CI). *P* 0.1, *I*^2^ ≤ 50%. If there was no heterogeneity between studies, a fixed-effect model was used. If heterogeneity existed, a random-effects model was used.

## Result

### Basic information of included studies

**Fig. 2:**
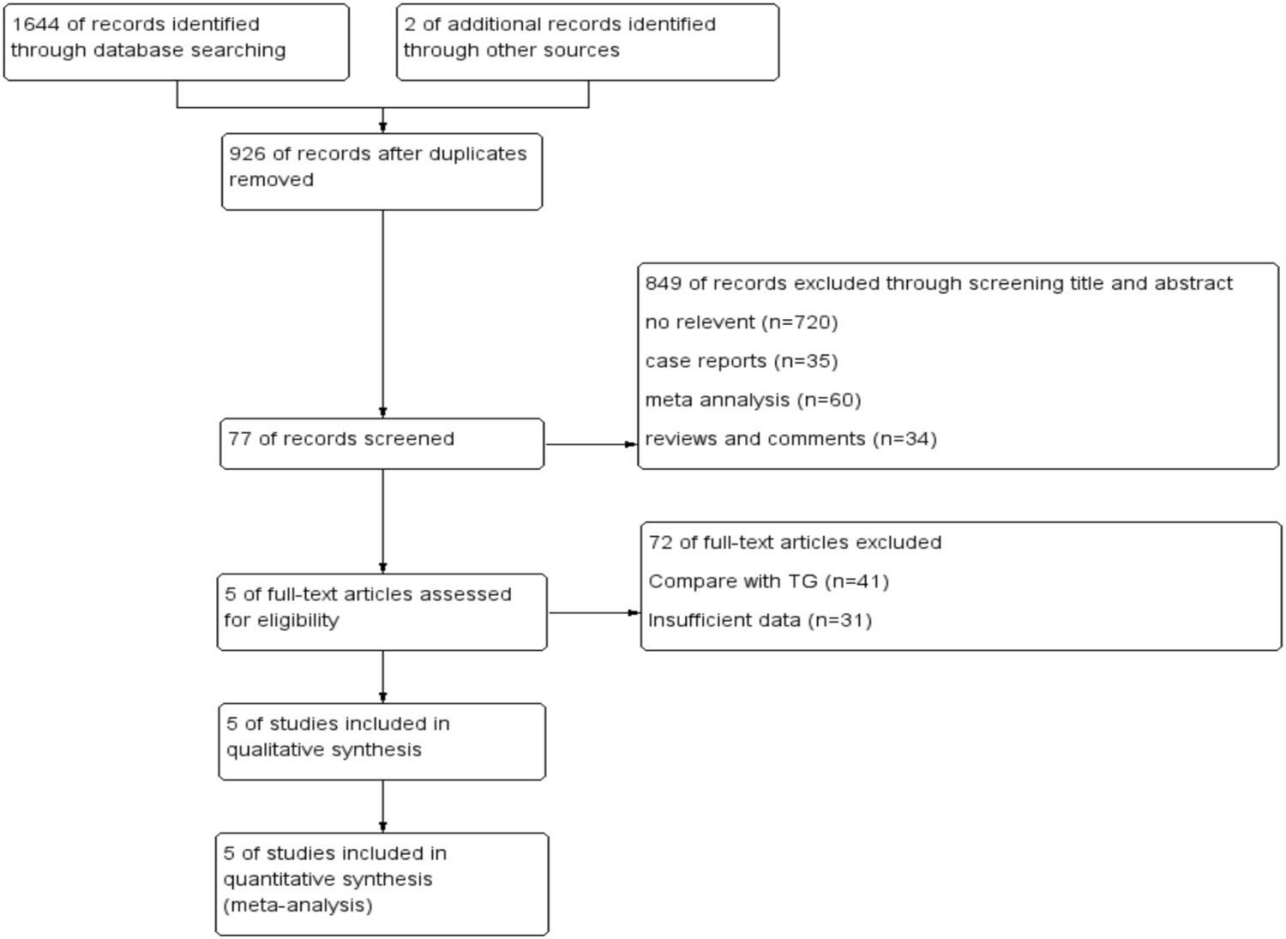
Flowchart of the study

A total of 1646 studies were retrieved, with 926 studies remaining after eliminating duplicate results. In addition, a total of 720 unrelated studies, 35 case reports, 60 meta-analyses, 34 reviews and commentaries, and 77 remaining studies were identified by searching the titles and abstracts of articles. Full-text retrieval, excluding 41 comparative studies with TG and 31 data studies that did not meet the requirements of this study, the remaining 5 were all included in the study (Fig. 2).

### Study characteristics

Two of the included studies were from Japan, Nishimura et al. [13] and Saze et al. [14], two studies from China, Wei et al. [15] and Li et al. [16], 1 study from Korea, Yu et al. [17] (Table 1). These studies included 409 patients (196 patients who received DFT and 213 patients who received DRT). The characteristics and NOS scores of the included studies are shown in Table 1.

### Preoperative situation

In the analysis of the basic patient profile, there were no significant differences in age, gender, and tumor stage between the two different surgical treatments. The results are listed in Table 2.

**Table 2:** Meta-analysis of preoperative basic data, perioperative situation, and partial nutritional status

Factor	No. of studies	No. of patients	Heterogeneity test		OR, WMD (95% CI)	*P*
			*I*^2^ (%)	*P*		
Age	5	409	17	0.31	0.53 (− 0.14 to 1.20)	0.12
Gender	5	409	0	0.70	0.87 (0.53 to 1.45)	0.60
Preoperative BMI	5	409	0	0.88	0.12 (− 0.10 to 0.35)	0.29
Preoperative Hb	3	298	19	0.29	5.92 (4.63 to 7.20)	< 0.0001
Perioperative complications	5	409	0	0.77	0.79 (0.44 to 1.42)	0.44
Operation time	4	359	94	< 0.0001	50.12 (5.70 to 94.53)	0.03
Surgical bleeding	3	180	96	< 0.0001	− 25.40 (− 66.37 to 15.56)	0.22
Pathological stage I	4	341	79	0.03	1.79 (0.25 to 12.84)	0.56
Reflux esophagitis	5	409	69	0.01	0.94 (0.13 to 6.67)	0.95
PPI intake	3	162	0	0.49	0.59 (0.22 to 1.59)	0.30
BMI after operation 1 year	2	248	0	0.75	2.01 (1.76 to 2.27)	< 0.0001
Hb after operation 1 year	3	298	58	0.09	4.26 (1.50 to 7.01)	0.002

### Surgical features

**Fig. 3:**
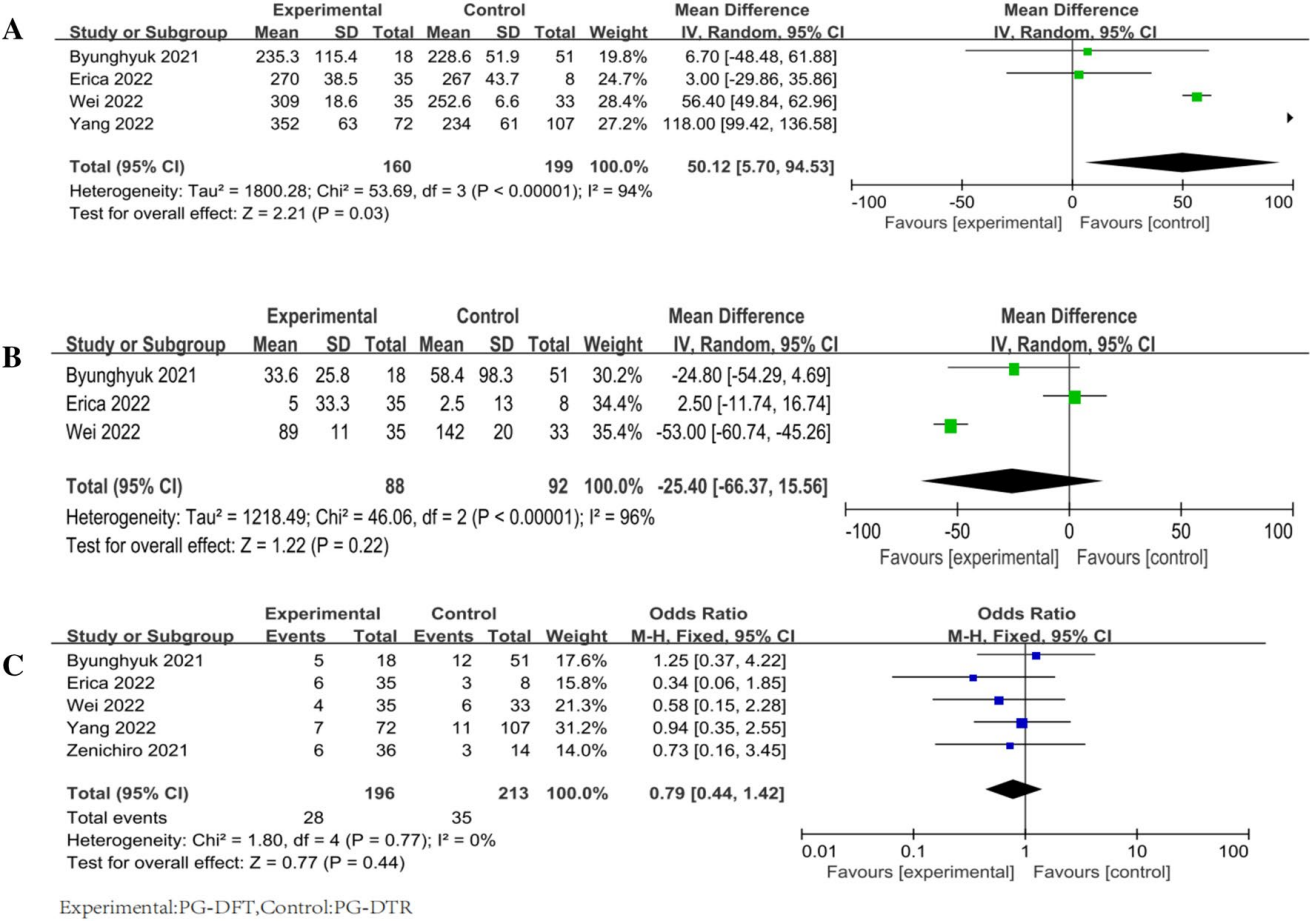
Forest plot of surgical features. **A** Operative time, **B** surgical bleeding, **C** perioperative complications

Four studies [13, 15–17] recorded operative time in patients with DFT (*n* = 160) and in patients with DTR (*n* = 199). The DTR group was shorter than the DFT group, and operative time was recorded in four of five studies, including DFT (*n* = 160), and DTR (*n* = 199). The difference was not statistically significant (*P* = 0.03) according to the random-effects model test (*χ*^2^ = 53.69, *P* < 0.00001; *I*^2^ = 94%) [*Z* = 1.22 (*P* = 0.03)]. (Fig. 3A).

Three papers [13, 15, 17] described surgical bleeding in patients with DFT (*n* = 88) and DTR (*n* = 92). There was no significant difference between DFT and DTR in terms of surgical bleeding. A random-effects model (*χ*^2^ = 46.06, *P* < 0.00001; *I*^2^ = 96%) [*Z* = 1.22 (*P* = 0.22)] was used (Fig. 3B).

All articles [13–17] reported perioperative complications, including DFT (*n* = 196) patients and DTR (*n* = 213) patients. A fixed-effects model [*Z* = 0.77 (*P* = 0.44)] was used because of strong homogeneity [*χ*^2^ = 1.80, *P* = 0.77; *I*^2^ = 0%)], and the results showed no significant difference between the two (Fig. 3C).

### Long-term complications

**Fig. 4:**
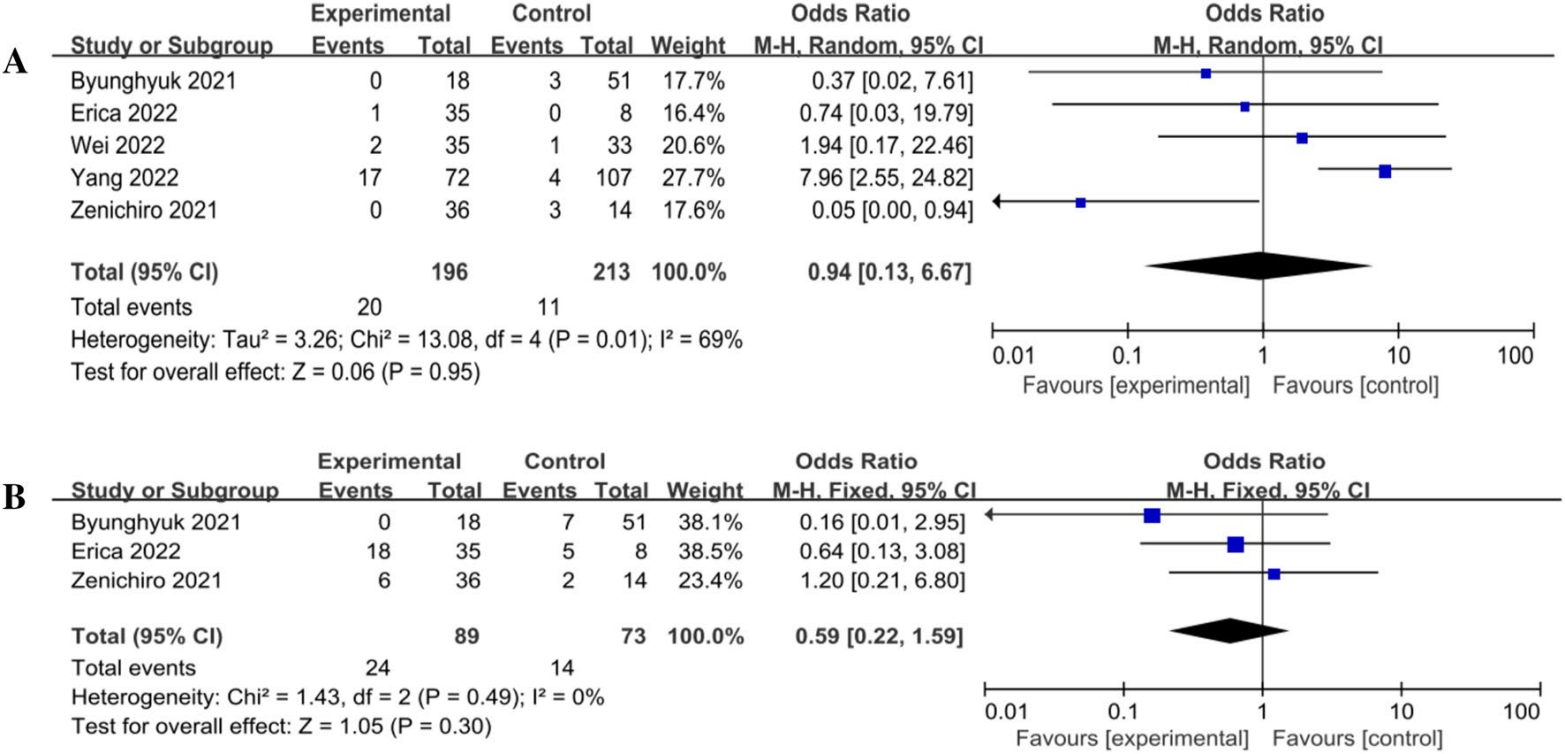
Forest plot of long-term complications. **A** Reflux esophagitis, **B** PPI intake

All studies [13–17] reported due to reflux esophagitis (*χ*^2^ = 13.08, *P* = 0.01; *I*^2^ = 69%), so we used random effect model for the study [*Z* = 0.06 (*P* = 0.95)]. The results showed no significant difference between DFT and DTR (Fig. 4A).

Three studies [13, 14, 17] reported PPI intake in patients with DFT (*n* = 89) and in patients with DTR (*n* = 73). A fixed-effects model was chosen for the analysis, because of (*χ*^2^ = 1.43, *P* = 0.49; *I*^2^ = 0%) [*Z* = 1.05 (*P* = 0.30)], and it showed no statistical significance in PPI intake between DFT and DTR (Fig. 4B).

### Partial nutritional status

**Fig. 5:**
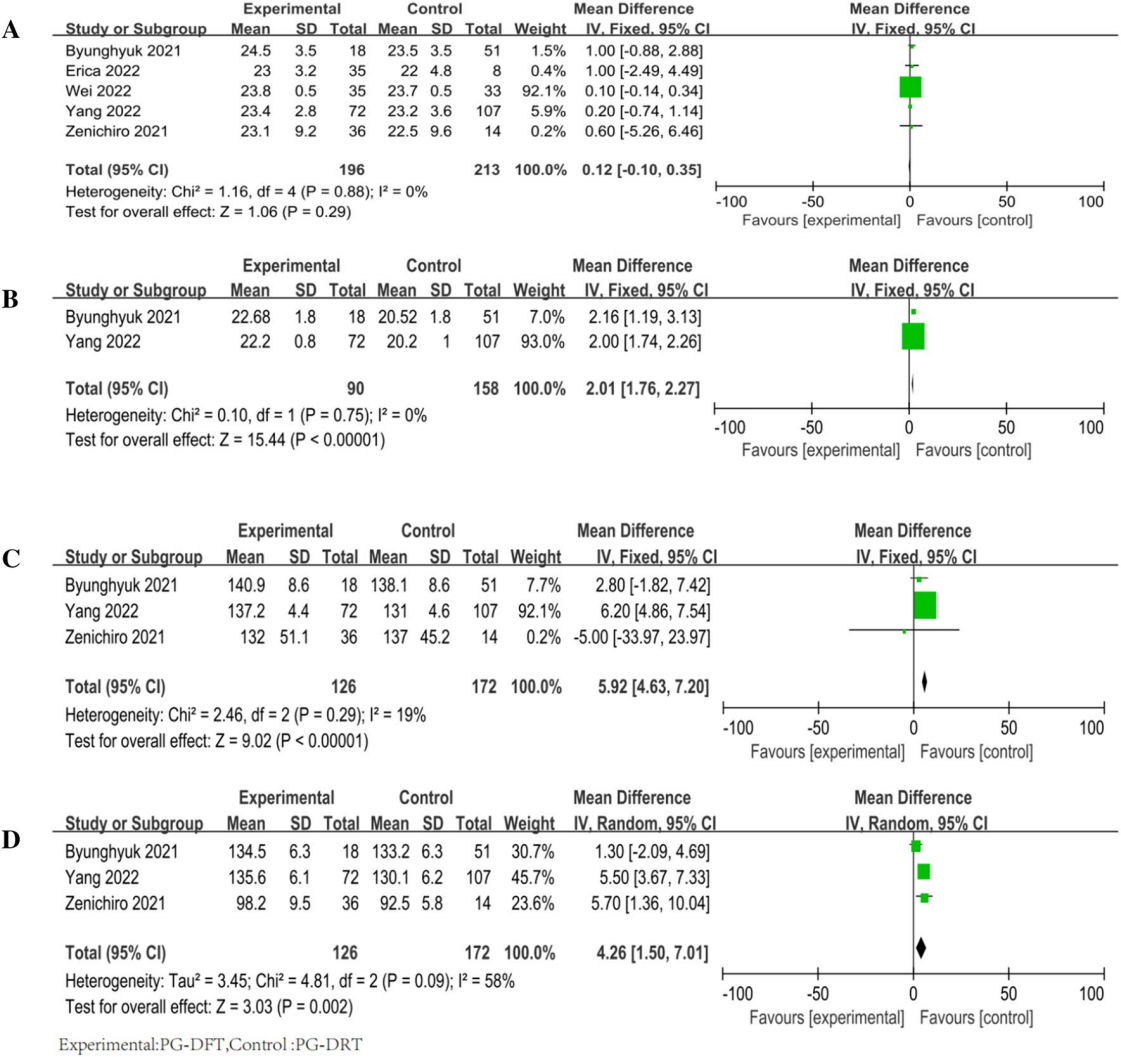
Forest plot of partial nutritional status. **A** Preoperative BMI, **B** BMI after operation 1 year, **C** preoperative Hb, **D** Hb after operation 1 year

All the studies [13–17] recorded preoperative BMI in patients with DFT (*n* = 196) and DTR (*n* = 213). However, only two studies [16, 17] included BMI at 1 year postoperatively. We observed no significant difference in preoperative BMI between DFT and DTR. We used fixed-effect model because of the strong homogeneity (*χ*^2^ = 1.16, *P* = 0.88; *I*^2^ = 0%) [*Z* = 1.06 (*P* = 0.29)] (Fig. 5A). However, the difference between DFT and DTR in BMI after operation 1 year was significantly (*P* < 0.0001). Fixed-effect model was selected for this meta-analysis (*χ*^2^ = 0.10, *P* = 0.75; *I*^2^ = 0%) [*Z* = 15.44 (*P* < 0.0001)] (Fig. 5B).

Only three studies [14, 16, 17] recorded both preoperative Hb and 1-year postoperative Hb, including patients with DFT (*n* = 126) and DTR (*n* = 172). Both were significantly different between DFT and DTR. Preoperative Hb was modeled using a fixed-effects model (CH2 = 2.46, *P* = 0.29; *I*^2^ = 19%) [*Z* = 9.02 (*P* < 0.0001)] (Figs. 2, 5c). In preoperative Hb, fixed-effect model was used (*χ*^2^ = 2.46, *P* = 0.29; *I*^2^ = 19%) [*Z* = 9.02 (*P* < 0.0001)] (Fig. 5C). In Hb after operation 1-year, random effect model was used (*χ*^2^ = 4.81, *P* = 0.09; *I*^2^ = 58%] [*Z* = 3.03 (*P* = 0.002)] (Fig. 5D).

### Publication bias assessment

**Fig. 6:**
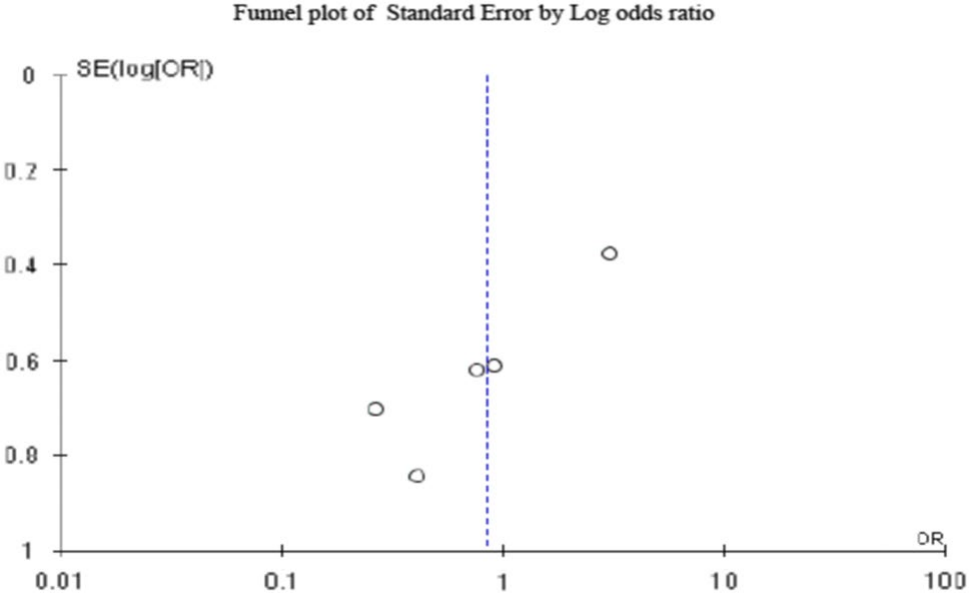
Symmetrical funnel plot with the majority of the studies present near the straight vertical line in the plot indicating no significant publication bias in the studies reviewed

The symmetry of the funnel plot (Fig. 6) with the majority of the studies present near the straight vertical line in the plot indicated no significant publication bias in the studies reviewed. According to the Egger’s regression test, the standard error was 0.34 and a two-tailed *P* value was 0.73 indicating no significant publication bias.

## Discussion

In this study, we systematically reviewed the studies of DFT and DTR, and performed a meta-analysis of five retrospective controlled clinical trials of DFT and DTR. As we know, this study is the first meta-analysis comparing the outcomes of DFT and DTR after PG. The focus was on the surgical outcome, the incidence of postoperative reflux esophagitis, and the postoperative nutritional status of the two reconstruction methods. We concluded that there was no significant difference between DFT and DTR in terms of age, gender, pathological stage, preoperative body mass index, intraoperative blood loss, and perioperative complications. However, the difference in operative time between the DFT and DTR groups was statistically significant (*P* = 0.03). This is because DFT anastomotic embedding requires manual suturing, whereas DTR has three anastomoses but is now basically anastomosed with an anastomosis and then manually reinforced. In our study, there was no significant difference between DFT and DTR in terms of reflux esophagitis and PPI uptake. The difference in Hb levels between preoperative and 1-year postoperative was not statistically significant, but the 1-year postoperative BMI results suggested that the nutritional status of DFT may be better than that of DTR 1-year postoperatively.

From the above results, DFT and DTR are similar and acceptable in terms of the feasibility and safety of surgery. However, postoperative nutritional status, especially postoperative weight changes, showed better results with DFT. Nishimura et al. [13] showed that DFT could protect subcutaneous and visceral fat even better than DTR, with even less need for anti-reflux medication. The results of Li et al. [16] and Saze et al. [14] also showed that the decrease in BMI at 1 year postoperatively in the DFT group was less than that of the DTR group, with a statistically significant difference. Yu et al. [17] found that the DTR group was more inclined to take anti-reflux medication for reflux symptoms than the DFT group (DTR 13.7% vs. DFT 0.0%, *P* = 0.177). The DTR group had a significantly lower body weight (*P* = 0.038) and body fat (*P* = 0.009). Although postoperative nutritional indicators were different and could not be compared by data, four of the five studies considered DFT to be associated with better postoperative nutritional status, apart from one of the five studies that lacked comparative data.

To parse these results, it is possible that all food in the postprandial DFT passes through the remnant gastroduodenal pathway into the jejunum, whereas in the DTR only part of the food passes through the remnant gastroduodenal pathway and the rest of the food passes through the middle jejunum pathway into the jejunum. Food that passes exclusively through the gastroduodenal route may have advantages in digestion and absorption. Takase et al. [34] concluded that the passage of food through the duodenal chyme helps to regulate the coordinated movement of the bile ducts and intestines, thereby stimulating the secretion of cholecystokinin and secretin, so that a variety of digestive fluids can enter the digestive tract and fully interact with each other to promote the absorption of nutrients. In summary, we believe that the most appropriate digestive tract reconstruction method should be selected according to the patient's specific situation. Compared with DTR, DFT reconstruction is technically demanding and time-consuming, but with better postoperative nutritional status, it should be used as the preferred GI reconstruction method for most patients with early proximal gastric cancer. However, DTR should be the best choice for patients with difficult surgery, intolerance to long-term surgery, and abnormal glucose tolerance [3].

The incidence of gastric cancer is among the top five in the world and mortality rate is among the top three [18]. Although the incidence of gastric cancer seems to be decreasing year by year, proximal gastric cancer is increasing year by year [19]. Proximal gastric cancer mainly includes gastroesophageal junction cancer or upper gastric cancer. Surgery is the main treatment for proximal gastric cancer, but the surgical strategy is still controversial. TG allows for more adequate tumor resection and lymphatic clearance. However, due to the removal of the entire stomach, the storage, mechanical grinding, and secretion functions of the stomach are lost, resulting in poor nutritional status and a high incidence of weight loss after surgery [20]. PG preserves the function of the remnant stomach and promotes the absorption of nutrients such as iron and vitamin B12. Compared with TG [21], PG improved the nutritional status and quality of life (QOL) of postoperative patients. Similarly, there is a high incidence of postoperative reflux esophagitis after PG due to retention of residual stomach. There have been many studies comparing PG and TG. Postoperative patients are well nourished and postoperative digestive tract reconstruction is relatively simple. The addition of anti-reflux measures during reconstruction can significantly reduce the incidence of postoperative reflux esophagitis in PG. TG is thought to have a better oncologic safety profile, but recent trials have reported no significant difference in overall survival between patients with early upper gastric cancer who underwent total and proximal gastrectomy [22]. Some studies have even shown that PG is superior to TG in terms of 5-year survival [21, 23]. In addition, PG is increasingly used in patients with PGC because it preserves some gastric function and the postoperative quality of life of patients is better than that of TG [24]. In 2018, the Japanese GC guidelines recommended PG as a surgical approach for early gastric tumors in the upper third [25]. According to the “Korea GC Practice Guide 2018” [26], PG is feasible for patients with type II and type III early AEG.

The anti-reflux anatomy of the esophagogastric junction consists mainly of the cardia and angle of His, but separation of the vagus nerve during proximal gastrectomy increases the incidence of pyloric spasm and impaired emptying of the remnant stomach [27]. Some patients may have postoperative reflux esophagitis with severe reduction in quality of life. Relevant studies suggest that the incidence of reflux esophagitis after PG may reach 50% [28]. To prevent the development of reflux esophagitis, several reconstructive procedures after PG have been reported, such as simple esophagogastrostomy, esophagogastrostomy with anti-reflux procedure, gastric tube formation, jejunal interposition, jejunal pouch interposition [29], DTR [30], and DFT [31]. DFT and DTR are the most used methods of digestive tract reconstruction after PG. The DTR reconstruction process is complex and requires three anastomoses so that food passes directly into the jejunum except through the remnant stomach, preventing reflux through the juxtaposition of the jejunum between the remnant stomach and the esophagus. In 2001, Kamikawa et al. [32] introduced DFT to prevent gastric acid reflux after PG. By encasing the muscle flap, a pseudo-cardia is formed to alleviate the reflux of gastric contents. Many previous studies have reported that double-serum muscle flap-enhanced anastomosis can reduce reflux esophagitis and anastomotic leakage after DFT [33]. However, DFT anastomosis requires manual suturing, which is a challenge for clinicians and adds much uncertainty. At present, several studies have reported that both surgical approaches have better postoperative nutritional status than TG with the same oncologic outcome. Meanwhile, both anti-reflux methods have achieved better results. There have been many studies comparing the prognosis of the two different reconstruction methods, but the current sample size is relatively small, multi-center, prospective comparisons are lacking, and the conclusions are controversial.

Although our results show that DFT may be the first choice for GI reconstruction in most patients with early proximal gastric cancer, there are still some limitations. First, only five studies were included in this meta-analysis, with a small total sample size. Second, the main comparative indicators, such as the selection and comparison of postoperative nutritional indicators, were different, resulting in less comparative data available for this study. The studies included in this study were retrospective studies with poor quality evidence. Finally, when comparing different surgical approaches, tumor safety is a priority. However, none of the included studies had long-term follow-up of survival. Future large multi-center prospective randomized controlled studies are needed to compare the role of DTR and DFT in long-term follow-up.

### Supplementary Information

Below is the link to the electronic supplementary material.

Supplementary file1 (DOCX 30 KB)

Supplementary file2 (DOCX 23 KB)

## Data Availability

The analytic dataset is available on request by contacting the corresponding author.

## Abbreviations

GC: Gastric cancer
TG: Total gastrectomy
PG: Proximal gastrectomy
DTR: Double-tract reconstruction
DFT: Double-flap technique
EBSCO: EBSCO Publishing
AEG: Adenocarcinoma of the esophagogastric junction
PG-DTR: Proximal gastrectomy with double-tract reconstruction
PG-DFT: Proximal gastrectomy with double-flap technique
Hb: Hemoglobin
PPI: Proton pump inhibitors
NOS: Newcastle–Ottawa Scale
CCT: Controlled clinical trial
WMDs: Mean weight difference
ORs: Odds ratio
CI: Confidence interval
QOL: Quality of life
BMI: Body mass index
