# Supplementary material for: Comparison of proximal gastrectomy with double-flap technique and double-tract reconstruction for proximal early gastric cancer: a meta-analysis
Source: Updates Surg. 2023 Sep 20;75(8):2117–26. doi: 10.1007/s13304-023-01638-w (PMC10710383; doi:10.1007/s13304-023-01638-w)
Supplement: Supplementary file 2 — Supplementary file2 (DOCX 23 KB) [file 13304_2023_1638_MOESM2_ESM.docx]

| **Section and Topic** | **Item #** | **Checklist item** | **Location where item is reported** |
| --- | --- | --- | --- |
| **TITLE** | | |  |
| Title | 1 | Identify the report as a systematic review. | Page 1, line 1-3 |
| **BACKGROUND** | | |  |
| Objectives | 2 | Provide an explicit statement of the main objective(s) or question(s) the review addresses. | Page 5-6, line 81-106 |
| **METHODS** | | |  |
| Eligibility criteria | 3 | Specify the inclusion and exclusion criteria for the review. | Page 7, line 125-133 |
| Information sources | 4 | Specify the information sources (e.g. databases, registers) used to identify studies and the date when each was last searched. | Page 6, line 111-113 |
| Risk of bias | 5 | Specify the methods used to assess risk of bias in the included studies. | Page 8-9, line 161-166 |
| Synthesis of results | 6 | Specify the methods used to present and synthesise results. | Page 9, line 168-175 |
| **RESULTS** | | |  |
| Included studies | 7 | Give the total number of included studies and participants and summarise relevant characteristics of studies. | Page 10 line185-190 |
| Synthesis of results | 8 | Present results for main outcomes, preferably indicating the number of included studies and participants for each. If meta-analysis was done, report the summary estimate and confidence/credible interval. If comparing groups, indicate the direction of the effect (i.e. which group is favoured). | Page10-12 line191-238 |
| **DISCUSSION** | | |  |
| Limitations of evidence | 9 | Provide a brief summary of the limitations of the evidence included in the review (e.g. study risk of bias, inconsistency and imprecision). | -Page 10, line 295-303 |
| Interpretation | 10 | Provide a general interpretation of the results and important implications. | Page 10, line 287-294 |
| **OTHER** | | |  |
| Funding | 11 | Specify the primary source of funding for the review. | Page 11, line 307-308 |
| Registration | 12 | Provide the register name and registration number. | Page 17, line 338-347 |

*From:*  Page MJ, McKenzie JE, Bossuyt PM, Boutron I, Hoffmann TC, Mulrow CD, et al. The PRISMA 2020 statement: an updated guideline for reporting systematic reviews. BMJ 2021;372:n71. doi: 10.1136/bmj.n71

For more information, visit: <http://www.prisma-statement.org/>
